# 3D culture increases pluripotent gene expression in mesenchymal stem cells through relaxation of cytoskeleton tension

**DOI:** 10.1111/jcmm.12946

**Published:** 2017-03-09

**Authors:** Ying Zhou, Haiyan Chen, Hong Li, Yaojiong Wu

**Affiliations:** ^1^ School of Life SciencesTsinghua UniversityBeijingChina; ^2^ The Shenzhen Key Laboratory of Health Sciences and TechnologyGraduate School at ShenzhenTsinghua UniversityShenzhenChina; ^3^ Tsinghua‐Berkeley Shenzhen Institute (TBSI)Tsinghua UniversityBeijingChina; ^4^ Department of General SurgeryQingdao Municipal Hospital QingdaoQingdaoChina

**Keywords:** mesenchymal stem cell, 3D, actin cytoskeleton, Nanog

## Abstract

Three‐dimensional (3D) culture has been shown to improve pluripotent gene expression in mesenchymal stem cells (MSCs), but the underlining mechanisms were poorly understood. Here, we found that the relaxation of cytoskeleton tension of MSCs in 3D culture was critically associated with the expressional up‐regulation of Nanog. Cultured in spheroids, MSCs showed decreased integrin‐based cell–matrix adhesion but increased cadherin‐based cell–cell interaction. Different from that in 2D culture, where MSCs exhibited branched and multiple‐directed F‐actin stress bundles at the cell edge and strengthened stress fibres transversing the cell body, MSCs cultured in spheroids showed compact cell body, relaxed cytoskeleton tension with very thin cortical actin filament outlining the cell, and increased expression of Nanog along with reduced levels of Suv39h1 (H3K9 methyltransferase) and H3K9me3. Notably, pharmaceutical inhibition of actin polymerization with cytochalasin D or silencing Suv39h1 expression with siRNA in 2D‐cultured MSCs elevated the expression of Nanog *via* H3K9 demethylation. Thus, our data suggest that 3D culture increases the expression of Nanog through the relaxation of actin cytoskeleton, which mediates reduced Suv39h1 and H3K9me3 levels.

## Introduction

Mesenchymal stem cells (MSCs) are expandable stem cells 1, 2, capable of differentiating into mesoderm and non‐mesoderm‐derived cells 1, 3, 4. However, previous studies indicate that in 2D culture conditions, MSCs suffer from several drawbacks, including increased cell size, decreased pluripotent genes expression such as *Nanog, Sox2, Oct4* with reduced self‐renewal ability, decreased production of paracrine factors and reduced capacity of engraftment and homing to injured tissues 4, 5, 6.

It has been well accepted that microenvironment has a pivotal role in stem cell fate decision. Apparently, conventional 2D culture fails to maintain a suitable microenvironment for MSCs. Recently, three‐dimensional (3D) culture methods have been adopted in MSC culture, which includes hanging‐drop‐based scaffold‐free system 4, 7, 8, stirring culture using spin flask or rotate wall vessel 9, 10 and cell aggregates formed on fabricated membrane 11, 12, 13, 14. Although involved with different culture devices, the pivotal event is with the cells; instead of sticking to the surface of culture dishes, cells aggregate to each other and form three‐dimensional spheres, which provide niches apparently different from those in 2D culture 15. Compared with 2D culture, 3D culture has been proved to benefit MSCs in several aspects, including reducing cell size potentially devoid of vascular obstructions 4, 7, 16, increasing self‐renewal and multipotent differentiation 4, 10, 12, 13, 14, 17, improving the capacity of engraftment and homing 7, 16, 18, the production of paracrine factors 7, 8, 9, and ultimate enhancing tissue repair in myocardial infarction 19, 20, brain stroke 21, angiogenesis 8 and wound healing 22.

Although the benefits of 3D culture to MSCs have been well demonstrated, the molecular mechanisms underlying were poorly understood. Here, we proposed that in hanging‐drop‐based 3D culture, which was scaffold‐free, changes of the niche were largely biophysics since the culture medium was the same as in 2D culture, and the system might serve as an ideal model to investigate the impact of mechanosensing on the alteration of MSCs property. With this model, we demonstrated that in 3D culture, integrin‐based cell–matrix adhesion decreased while cadherin‐based cell–cell interaction increased. This led to reduced expression of β‐actin with decreased cell size on one hand, and increased Nanog expression on the other hand, (at least) in part through the reduction in Suv39h1 and H3K9me3 accumulation.

## Materials and methods

### Cell isolation and culture

Human MSCs were isolated from human placenta as described previously 5. Briefly, term (38–40 weeks' gestation) placentas from healthy donors were harvested with written informed consent and the procedure was approved by the Ethics Committee of Xili Hospital. The placental tissue was washed several times with cold PBS and then mechanically minced and enzymatically digested with 0.25% trypsin‐ethylenediaminetetraacetic acid (EDTA) for 30 min. at 37°C in a water bath. The digest was subsequently filtered, pelleted and re‐suspended in a growth medium consisting of DMEM (Gibco‐Invitrogen, Carlsbad, CA, USA), 10% foetal bovine serum (FBS; Gibco‐Invitrogen) and antibiotics. Cells were seeded on polystyrene dishes, and medium was replaced every 2 days to reach 80% confluence. Cells were subcultured after trypsinization. To form spheroids, passage 5–7 MSCs were cultured by a hanging‐drop method as described previously 7, with modifications. Briefly, MSCs were plated in hanging drops in 35 μl DMEM containing 10% FBS and 20,000 cells per drop and incubated for 36 hrs. Then, the spheroids were transferred to a suspension culture and incubated in fresh growth medium for 24 hrs. To obtain single cells from spheroids, spheroids were incubated with 0.25% trypsin/EDTA for 4–6 min. with gentle pipetting every 2–3 min. 4. For visualization of nuclear actin filament, MSCs transfected with LifeAct‐GFP‐NLS were handled as usual subculture. For 2D MSCs, cell suspension was plated on to glass bottom well (NEST). For 3D MSCs, coating the glass bottom well with 1% agarose (Gene Company Ltd, Hongkong, China) before cell suspension was plated and MSCs formed sphere spontaneously.

### Real‐time PCR analysis

Total RNA was extracted from MSCs with TRIzol (Invitrogen, New York, America) following the manufacturer's instructions. First‐strand cDNA was prepared by reverse transcription with Superscript II reverse transcriptase (Invitrogen) and oligo (dT) primers and stored at −20°C. Real‐time PCR was performed with SYBR Green Real‐Time PCR Master Mix (TOYOBO, Osaka, Japan) on an ABI 7300 QPCR System. As an internal control, levels of glyceraldehyde‐3‐phosphate dehydrogenase (GAPDH) were quantified in parallel with target genes. Normalization and fold changes were calculated using the ΔΔCt method. Primer sets are as follows: *Nanog*: 5′‐AATACCTCAGCCTCCAGCAGATG‐3′; 5′‐TGCGTCACACCATTGCTATTCTTC‐3′; β*‐actin*: 5′‐CATGTACGTTGCTATCCAGGC‐3′; 5′‐CTCCTTAATGTCACGCACGAT‐3′; *Suv39h1*: 5′‐CCTGCCCTCGGTATCTCTAAG‐3′; 5′‐ATATCCACGCCATTTCACCAG‐3′; *Suv39h2*: 5′‐TCTATGACAACAAGGGAATCACG‐3′; 5′‐GAGACACATTGCCGTATCGAG‐3′; *G9A*: 5′‐AAAACCATGTCCAAACCTAGCAA‐3′; 5′‐GCGGAAATGCTGGACTTCAG‐3′; *SETDB1*: 5′‐TAAGACTTGGCACAAAGGCAC‐3′; 5′‐TCCCCGACAGTAGACTCTTTC‐3′; *OCT4*: 5′‐GTGTTCAGCCAAAAGACCATCT‐3′; 5′‐GGCCTGCATGAGGGTTTCT‐3′; *DPPA3*: 5′‐TAGCGAATCTGTTTCCCCTCT‐3′; 5′‐CTGCTGTAAAGCCACTCATCTT‐3′; *ERAS*: 5′‐GAGTCTGGCGGAAAAGCTGTA‐3′; 5′‐GGTGCGGAATGTCATCGTAGG‐3′; *NR5A2*: 5′‐CTCTGACCTGACCATTTCCTCT‐3′; 5′‐TGTCATAGTCTGTAGGAGGCAAG‐3′.

### Immunofluorescence

Human MSCs grown on coverslips were fixed in 1% PFA (Polyformaldehyde). Human MSC spheroids were fixed in 3% PFA, embedded in OCT and cryosectioned (6–8 μm thickness). After permeabilization with 0.1% Triton X‐100, samples were incubated with desired primary antibodies, overnight at 4°C, followed by detection with a fluorescence‐conjugated secondary antibody. Nuclei were stained with 4, 6‐diamidino‐2‐phenylindole (DAPI). After mounting, samples were visualized under confocal microscope (FV1000; Olympus, Tokyo, Japan). Nuclear actin was observed directly. Confocal z‐scans were used and display an overlay of several picture 23.

F‐actin was labelled with Alexa Flura488‐phalloin (1:400; Life Technologies, Carlsbad, CA, USA); G‐ actin was labelled with Rhodamine‐DNase I (1:1000; Life Technologies); other antibody used in this study were α‐tubulin (1:400; Millipore, America); β‐actin (1:100; Santa Cruz, America); Nanog (1:200; ThermoFisher, America); Suv39h1 (1:200; Millipore, America); H3K9me3 (1:500; Abcam); Integrin β1 (1:100; Millipore); Vinculin (1:200; Santa Cruz); N‐cadherin (1:400; Cell Signaling Technology); β‐catenin (1:400; Sigma‐Aldrich); focal adhesion kinase (FAK, 1:1000; Millipore); phosphorylated FAK (p‐FAK, 1:1000; Millipore, America); E‐cadherin (1:2000; Life Technologies, America).

### Transfection

LifeAct‐GFP‐NLS was a gift from Dr. Wang Sheng. Passage 3 MSCs were used for transfection. Mesenchymal stem cells of passage 5–7 were used in small‐interfering RNA (siRNA) transfection. Transfection with Neon Transfection System (Invitrogen, America), as manufacturer's instruction. Briefly, Target MSCs for transfection were cultured in antibiotic‐free CCM and harvested as described above. About 1 × 10^6^ MSCs were resuspended in 100 μl resuspension buffer R (Neon Transfection System; Invitrogen) with 1 μM siRNA or 5 μM plasmid and transfected in 100 μl Neon tip with Neon transfection system (Invitrogen) using two pulses (1400 V input pulse voltage/20 msec. input pulse width). Transfected MSCs were plated on 10 cm^2^ tissue culture dishes in 10 ml antibiotic‐free CCM for 24 hrs before further treatment 24. Targeted sequences were si *Suv39h1* 5′‐CGUGGAUUGUCUCAGGGAATT‐3′; 5′‐UUCCCUGAGACAAUCCACGTGTT‐3′ (Gene Pharm, Hong Kong, China).

### Chromatin immunoprecipitation

Chromatin immunoprecipitation (ChIP) assay was performed with passage six MSCs, as previously described 4, according to the fast ChIP method 25, except that the protein‐A sepharose was replaced by protein G Dynabeads (Life Technologies) and that the antibodies (against H3; Cell Signaling Technology and H3K9me3; Abcam) were incubated with samples overnight at 4°C. Normal rabbit IgG (Millipore) was used for mock IP. The purified DNA was measured by quantitative fluorescent PCR analysis using SYBR Green real‐time PCR Master Mix (TOYOBO) on an ABI 7300 QPCR System. Primer sets were as follows: N1: 5′‐GTTCTGTTGCTCGGTTTTCT‐3′; 5′‐TCCCGTCTACCAGTCTCACC‐3′; N2: 5′‐CACCTATGCCTGTGATTTGTG‐3′; 5′‐AACCCTCTCTCCTTCTCTCTTTTC‐3′; N3: 5′‐GCCCTATCCAAATCCTATCACTT‐3′; 5′‐GGTCAGCACAAAATACAGGTCA‐3′.

Data were analysed using Percent Input Method (Life Technologies), in which equal amounts of starting chromatin were used as input, and signals obtained from the ChIP over the background were divided by signals obtained from an input sample. The results were counted with the formula: percent input = 100%*(2 ^Ct (input)‐Ct (antibody)^ − 2^Ct (input)‐Ct (mock)^).

### Flow cytometry

Passage 6 cells were resuspended in PBS containing 1% bovine serum albumin at 10^6^/ml; 100 μl cell aliquots were incubated with β‐catenin (1:200; Sigma‐Aldrich) or control isotype IgG on ice for 30 min., washed with PBS twice and detected with a fluorescence‐conjugated secondary antibody. Ten thousand events were analysed by flow cytometry (Becton Dickinson, America) using Cell Quest software.

### Preparation of cell fractions and immunoblotting

Total cell lysates were prepared by incubation of cells in RIPA lysis buffer containing protease inhibitors (Roche, America). For nuclear fraction, single‐cell suspensions of MSCs cultured in regular 2D condition or in 3D spheroids were obtained by trypsinization; washed with cold PBS and centrifuged at 1000 × g for 5 min. The cell pellet was kept at −80°C for 45 min., before resuspension in buffer P1 Hepes 10 mM, EGTA 0.1 mM, DTT 1 mM, complete protease inhibitors (Roche). After addition of Triton X‐100 (final concentration 0.5%), samples were vortexed for 10 sec., followed by sedimentation of nuclei at 10,000 × g for 10 min. The nuclear pellet was washed with buffer P1 before lysis in buffer P2 (Hepes 20 mM, Glycerol 25%, NaCl 400 mM, EGTA 1 mM, DTT 1 mM, complete protease inhibitors) for 90 min. on a rotary shaker at 4°C. Remaining insoluble material was sedimented at 16,000 × g for 30 min. The purity of obtained extracts was controlled by immunoblotting for GAPDH and histone H3 23. Membrane Protein Extraction Kit (ThermoFisher) was used for membrane protein extraction. 2D MSCs were handled as adherent mammalian cell, while equal amount of 3D spheres was operated as soft tissue as per manufacturer's instruction. Before membrane extraction, a fraction of cell from 2D and 3D MSCs were subjected to total cell lysis. Immunoglobulin for GAPDH with total cellular protein from this step was used as loading control.

### Statistical analysis

All data were expressed as mean ± S.E.M. for the number of samples indicated (*n*). One‐way anova was used for data analysis and statistical significance was defined as *P* < 0.05.

## Results

### 3D culture changes the expression of adhesion molecules in MSCs

**Figure 1:**
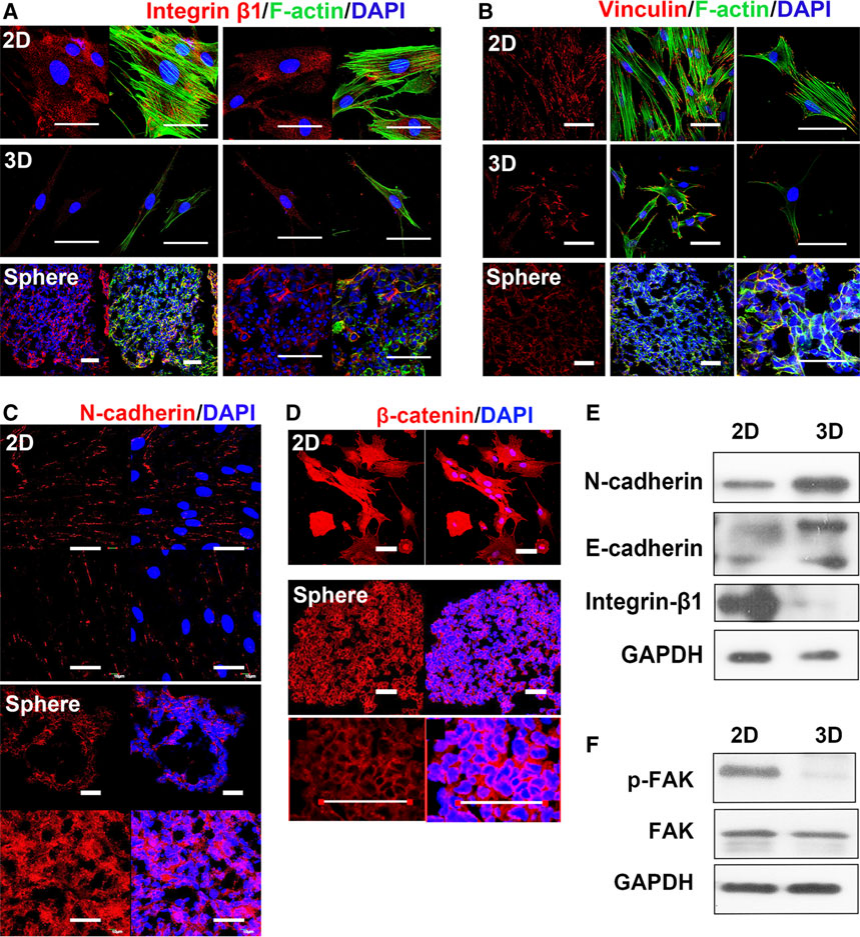
Adhesion molecules on the membrane of 2D and 3D MSCs. Membrane proteins in MSCs were detected with immunostaining for the indicated material. 2D: MSCs cultured as monolayer on slide, cultured overnight before fixation. 3D: MSCs, after 3D spheroid cultured for 60 hrs, trypsinized and re‐plated on slide, cultured overnight before fixation. 3D sphere: 10–20 μm of frozen section of fixed sphere, to analyse MSCs *in situ*. Immunostaining of integrin β1 (**A**) and Vinculin (**B**) were presented in red. F‐actin was labelled by phalloidin (Alexa‐Fluor 488‐conjugated, green). Cadherin‐based connection is established mostly at the cell–cell contact, so only confluent 2D MSC and 3D sphere were used to stain N‐cadherin (**C**) and β‐catenin (**D**). Red, antibody staining; blue, DAPI staining (scale bar, 50 μm). (**E**) Membrane fraction of 2D and 3D MSC were immunoblotted for N‐cadherin, E‐cadherin and integrin β1. Before membrane extraction, a fraction of cell from 2D and 3D MSCs were subjected to lysis. Immunoblotting for GAPDH with total cellular protein from this step was used as loading control. (**F**) Total cellular protein of 2D and 3D MSCs immunoblotting for FAK, phosphorylated FAK (p‐FAK) and GAPDH (loading control). These data are representative of five independent experiments with similar results.

It is known that mechanical properties of the surrounding microenvironment affect the fate of MSCs, which are sensed by adhesion complex on the cell membrane, and conducted by actin cytoskeleton tension 26. Compared with stiff substrates in 2D culture, the physical feature of microenvironment in 3D MSCs was different 15. To understand the influence of culture conditions on the adhesion molecules, we first determined the expression of adhesion proteins. Cultured as 2D, abundant integrin β1 was found on the membrane of 2D MSCs, together with massive Vinculin distribution, resulting in highly contraction of attached cytoskeleton, while in 3D MSCs, the levels of integrin β1 and Vinculin declined markedly (Fig. 1A and B). The remarkable reduction of integrin signalling in 3D MSCs was confirmed by the level of p‐FAK as determined by Western blot analysis (Fig. 1F). In addition, cultured in 3D spheres, cell–cell interaction increased, which was associated with an up‐regulation of N‐cadherin and E‐cadherin (Fig. 1C and E). β‐catenin is known to connect and stable the intracellular domain of cadherins on the membrane 27. We found that β‐catenin was distributed across the cell in 2D MSCs, while it was accumulated on the surface in 3D MSCs (Fig. 1D). Flow cytometry analysis indicated that β‐catenin was expressed in the majority (95%) of both 2D and 3D MSCs (Fig. S1).

### 3D culture alters the cytoskeleton of MSCs

**Figure 2:**
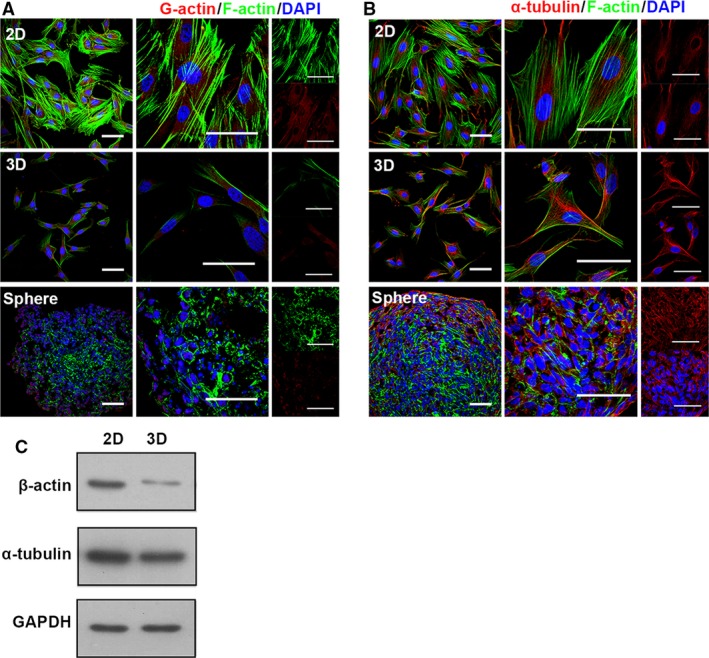
Cytoskeleton altered in 3D‐cultured MSCs. Two kinds of typical cytoskeleton in MSC were detected with immunostaining for the indicated material (abbreviation as in Fig. 1). (**A**) F‐actin was visualized using phalloidin (Alexa‐Fluor 488‐conjugated, green), G‐actin using DNase I (Rhodamine‐conjugated, red), higher magnification images were shown in right panel. (**B**) To show the structure of microtubule, α‐tubulin (red) was immunostained, higher magnification images were shown in right panel. Nuclei were labelled using DAPI (blue) (scale bar, 50 μm). (**C**) Total cellular protein of 2D, 3D MSCs immunoblotting for α‐tubulin, β‐actin, GAPDH (loading control). These data are representative of five independent experiments with similar results.

After being cultured in 3D spheroids for 60 hrs, MSCs were trypsinized, dissociated and re‐adhered to culture plates, resulting in uniformed spindle‐like cells with the characters of smaller size and less spreading with sharp edges 4, 28. The cytoskeleton supports cell morphology according to the mechanical and biochemical properties of microenvironment 29. We explored the organization of β‐actin and α‐tubulin, which polymerizes to form main types of cytoskeleton filaments in eukaryotes: microfilaments and microtubules. Adapted to spheroid culture, MSCs adopted a different pattern of cytoskeleton organization (Fig. 2). In 2D MSCs, cytoskeleton of both types formed stretch out networks, in lined with the flat morphology, while in 3D culture, the cytoskeleton was loose in tension. Different from the organization of α‐tubulin, the organization of actin cytoskeleton in 3D culture remained after trypsinized and cultured as monolayer (Fig. 2B). As shown in Figure 2A and D, MSCs show branched and multiple‐directed F‐actin stress bundles at the cell edge as well as transversing the cell body, resulting in big and flat 2D MSCs. In contrast, cells in 3D spheroids demonstrates cortical actin filament outlining the cell and very thin filament crossing the cell body, and the filaments are mostly lined in parallel, which contributes to the smaller and uniformly spindle‐like morphology (Fig. 2A).

We examined the protein level of both cytoskeleton monomeric molecules in MSCs after 3D culture; the amount of α‐tubulin remained unchanged, while the level of β‐actin reduced dramatically, which may contribute to the long‐lasting alteration in the arrangement of actin cytoskeleton (Fig. 2C). The results suggest that the re‐arrangement of actin cytoskeleton is largely responsible for the lasting impact of 3D culture on cell size and morphology.

### 3D culture releases actin cytoskeleton tension in MSCs

**Figure 3:**
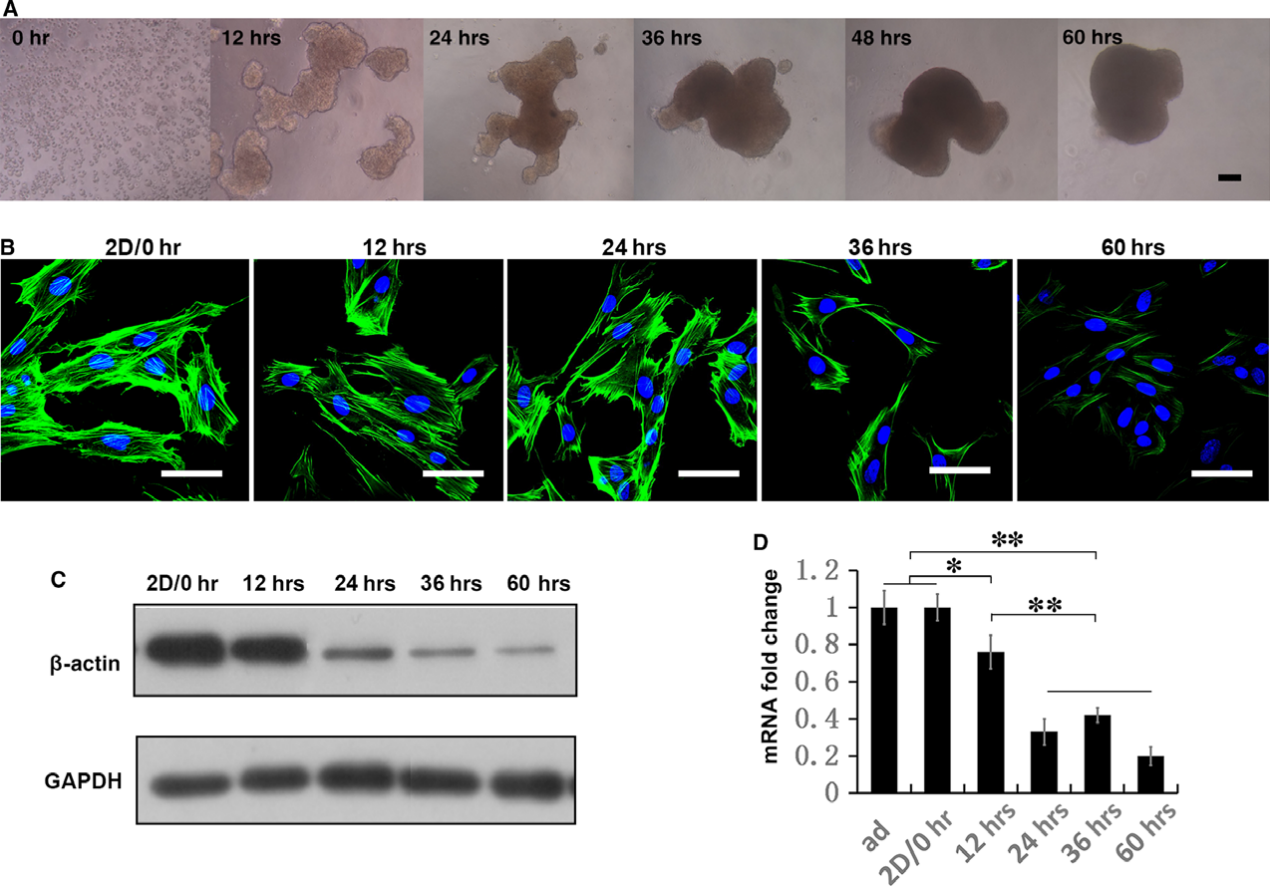
Actin‐cytoskeleton released gradually in 3D MSCs. (**A**) Microscope images show the process of sphere formation in 60‐hr duration. (**B**) Cultured in 3D for different duration, MSCs were trypsinized and re‐plated as monolayer and assessed for F‐actin arrangement with phalloidin (Alexa‐Fluor 488‐conjugated, green). Actin cytoskeleton restores the mechanism response in 3D culture, so MSCs derived from different time‐points in 3D culture presents continuous variation in F‐actin stress and arrangement (scale bar, 50 μm). Protein amount of β‐actin in 2D and 3D MSCs were analysed by Western blotting (**C**). These data are representative of three independent experiments with similar results. (**A**–**C**) Expression of β‐actin in 2D and 3D MSCs were analysed by real‐time PCR (**D**). Data are mean ± S.E.M. (*n* = 3); **P < 0.01 (Duncan's multiple range test).

Cultured in hanging drops, MSCs formed spheroid gradually. In the first 12 hrs, dispersed MSCs in suspension contacted and formed several small and loose aggregates, then the aggregates grew by further fusion and compaction, until all the cells were assembled together. This took 36 hrs in general and it would take another 24 hrs for the aggregates to re‐shape and form a regular spheroid (Fig. 3A). To investigate the long‐lasting actin cytoskeleton alteration in this process, we analysed MSCs at these time‐points. The results showed that the protein and mRNA levels of β*‐*actin declined slightly in the first 12 hrs, while the F‐actin stress fibre also decreased slightly in density, mostly at the cell edge, as determined in the re‐adherent cultured cells. The major reduction in the expression of β*‐*actin took place in the second 12 hrs, which was associated with a decline both in the level of F‐actin stress fibre and the cell size. In the last 36 hrs, the protein level of β‐actin and the level of stress fibre reduced further, resulting a remarkably altered cell morphology and actin pattern in 3D MSCs (Fig. 3 B–D).

### Nuclear actin filament remains assembled in 3D MSCs

**Figure 4:**
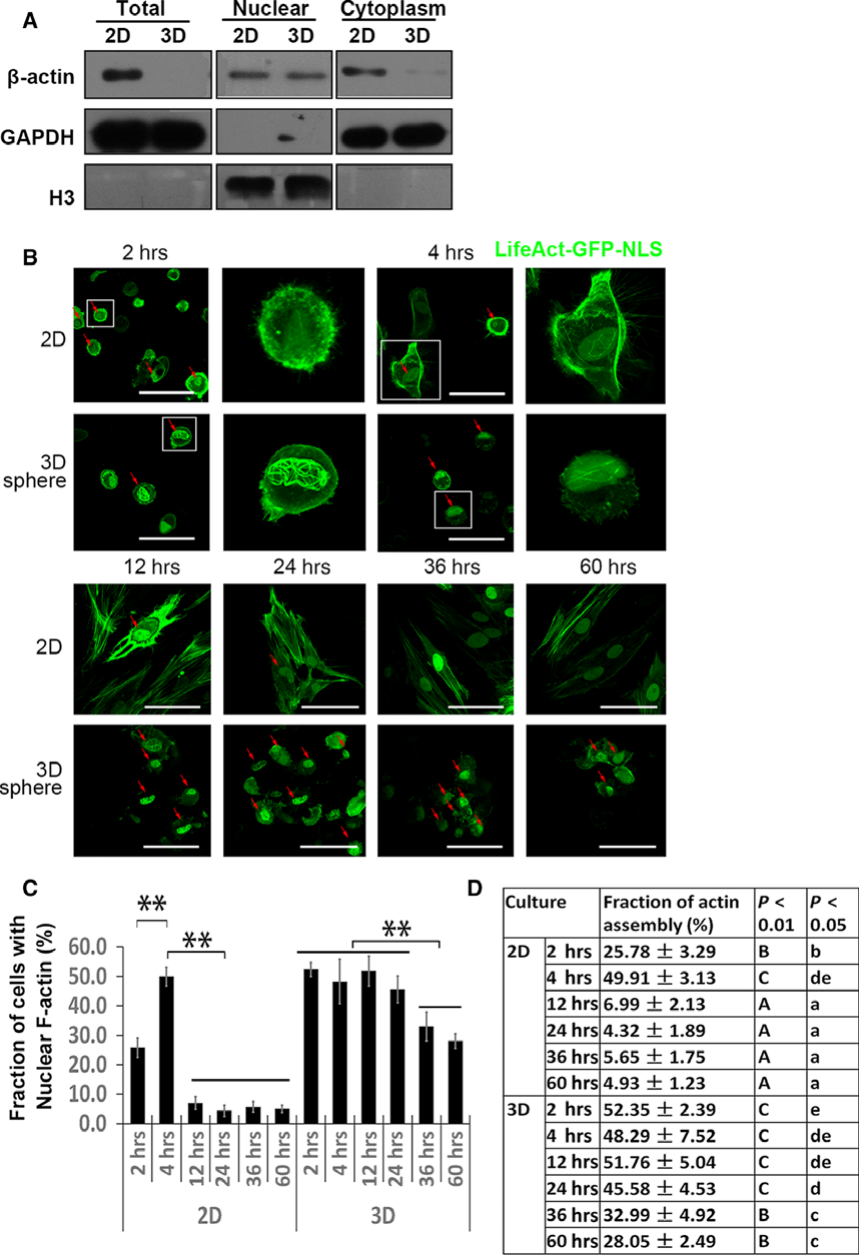
Temporal characterization of nuclear F‐actin formation during 2D and 3D culture. (**A**) Subcellular fractions were immunoblotted for β‐actin, GAPDH (cytoplasm loading control) and histone H3 (nuclear loading control). LifeAct‐GFP‐NLS was transfected to visualize nuclear F‐actin formation. MSCs were plated on culture dish as monolayer or dispersed in suspension to form spheres spontaneously and were monitored over time. Individual frames show F‐actin assembly at indicated time‐points (**B**). Red arrowheads mark MSCs with nuclear F‐actin formation. Higher magnification images of selected MSCs with typical nuclear F‐actin were shown. Quantification of nuclear F‐actin formation during culture duration as a fraction of cells with nuclear F‐actin over GFP positive cells (**C** and **D**) (scale bar, 50 μm). Four experiments were independently performed, and each time, over 50 cells were counted for each time‐point. Uppercase letters refer to *P* < 0.01, lowercases refer to *P* < 0.05 (Duncan's multiple range test).

Filamentous actin in the nucleus was considered necessary for transcriptional re‐activation of pluripotent genes 30. Immunoblotting of β‐actin in the subcellular fraction of 3D MSCs showed a dramatic decline of cytoplasmic β‐actin, while only a slight decrease in nuclear β‐actin (Fig. 4A). With the relatively increased proportion of nuclear actin, we proposed that F‐actin might form in the nucleus to support the nuclear structure in 3D MSCs. To visualize nuclear actin in MSCs, we generate MSCs with transient expression of Lifeact‐GFP‐NLS. Of note, compared with NIH 3T3 31, spreading‐induced nuclear actin filaments appeared later in MSCs (Fig. 4B). We subsequently analysed and quantified nuclear actin assembly in 2D and 3D MSCs. In 2D MSCs, nuclear F‐actin was detectable in 1–2 hrs after plating and attained the maximum response after 4 hrs, then slowly declined (Fig. 4 B–D). After 12 hrs, only a fraction of 2D MSCs (<7%) displayed nuclear F‐actin formation. In contrast, nuclear F‐actin assembly in 3D MSCs was detectable after 2 hrs, with a higher positive rate at about 50%, and lasted for 24 hrs. After 24 hrs, the diffused fluorescent signal of LifeAct‐GFP‐NLS declined in general, which might attribute to the slight declination of the positive rate of nuclear F‐actin assembly at 36 and 60 hrs.

### 3D culture restores Nanog expression and H3K9 demethylation in MSCs

**Figure 5:**
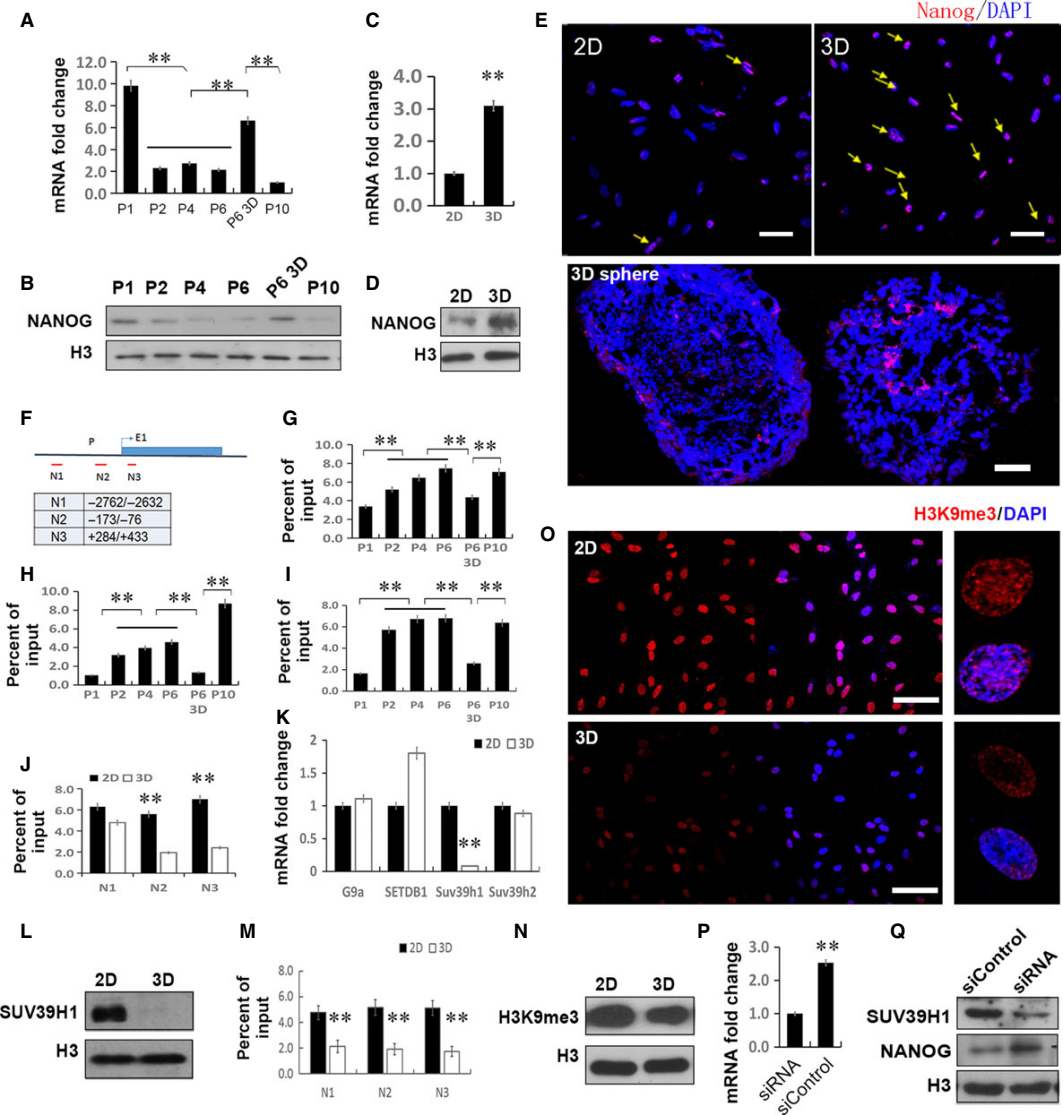
Restored Nanog expression and H3K9 demethylation in 3D MSCs. (**A**) Real‐time PCR and (**B**) Western blotting showed Nanog expression down‐regulated progressively once passaged in 2D method and increased after 3D cultured. **P < 0.01 (Duncan's multiple range test), three samples were used each time, and three independent experiment were performed and yield similar results. (**C**) Real‐time PCR analysis of Nanog expression in 2D and 3D MSCs. **P < 0.01 (*n* = 3). (**D**) Nuclear faction of 2D and 3D MSCs was immunoblotted for Nanog and histone H3 (nuclear loading control). The data are representative of three independent experiments with similar results. (**E**) Nanog expression patterns in 2D, 3D and 3D sphere MSCs (abbreviation as Fig. 1). Red, antibody staining; blue, DAPI staining. Yellow arrowheads mark MSCs positive for Nanog (scale bar, 50 μm). The data are representative of three independent experiments with similar results. (**F**) Regions examined by ChIP. (**G**–**I**) H3K9me3 accumulation level evaluated by ChIP‐qPCR on region of N1 (**G**), N2 (**H**), N3 (**I**) at indicated passage of MSCs. (**J**) H3K9me3 ChIP‐qPCR assay in 2D and 3D MSCs. Three samples were used each time, and three independent experiment were performed and yield similar results. Duncan's multiple range test was used in (**H**–**J**). (**K**) Real‐time PCR analysis of methyltransferases responsible for H3K9me3 level. **P < 0.01 (*n* = 3). (**L**) Western blotting of Suv39 h1 in nuclear fraction. (**M**) Abundance of Suv39 h1 on Nanog promoter was assessed by ChIP. (**N**) The effects of 2D and 3D culture on H3K9 methylation levels. Chromatin histone was extracted from 2D and 3D MSCs. (**O**) H3K9me3 accumulation patterns in 2D and 3D MSCs. Red, H3K9me3 staining; blue, DAPI staining (scale bar, 50 μm). Higher magnification images were shown in right panel. These data are representative of three independent experiments with similar results. (**P**,** Q**) Knockdown of by siRNA restored Nanog expression. **P < 0.01 (*n* = 3), Duncan's multiple range test.

Cultured in 2D, MSCs showed decreased expression of Nanog at both mRNA (Fig. 5A) and protein levels (Fig. 5B).

As described in our previous paper 4, when cultured in 3D condition, Nanog expression increased as determined by real‐time PCR analysis (Fig. 5C) and Western blot (Fig. 5D). In addition, immunostaining barely detected the expression of Nanog in 2D MSCs, but evidently revealed the expression of the protein in the nucleus of numerous 3D‐cultured MSCs (Fig. 5E).

Contiguous H3K9me3 enrichment triggered by the culture environment is the major barrier in Nanog expression in somatic cells 32, 33. To determine the relation between Nanog expression and H3K9me3 levels, H3K9me3 was immunoprecipitated by ChIP and its enrichment relative to input chromatin was assessed by qPCR with three pairs of primers focusing on the promoter region of Nanog (Fig. 5F). We found that associated with a decline in Nanog expression in 2D‐cultured MSCs upon passaging, H3K9 tri‐methylation accumulated in the promoter of Nanog, especially in regions near the transcription starting site (Fig. 5G–I). Moreover, in accordance with the increase of Nanog expression in 3D MSCs, the H3K9me3 occupancy decreased (Fig. 5J).

To explore the mechanisms underlying H3K9 tri‐methylation reduction, candidate genes of methyltransferases responsible for H3K9 tri‐methylation accumulation were screened. Among them, Suv39h1 expression significantly reduced at the mRNA level (over sixfold, *P* < 0.01) and protein level (Fig. 5K and L). The enrichment of Suv39h1 on the promoter of Nanog also reduced in 3D MSCs (Fig. 5M), thus confirming Suv39h1 mediating H3K9me3 levels. As Suv39h1 has been known to be responsible for the H3K9me3 abnormally accumulation and heterochromatin aberrantly increase in stem cell senescence 33, 34, 35, we determined the global level of H3K9me3. Western blotting and immunofluorescence showed that in 3D MSC, H3K9me3 level was lower and distributed diffusely at the nuclear periphery, while in 2D MSCs, H3K9me3 clustered into dense foci that co‐localized with the nucleus (Fig. 5N and O). Knocking down Suv39h1 with a siRNA enhanced Nanog expression in MSCs, suggesting that the reduction of Suv39h1 may also be responsible for the up‐regulation of Nanog expression in 3D MSCs (Fig. 5P and Q).

### Release of actin cytoskeleton tension promotes H3K9 demethylation and Nanog expression

**Figure 6:**
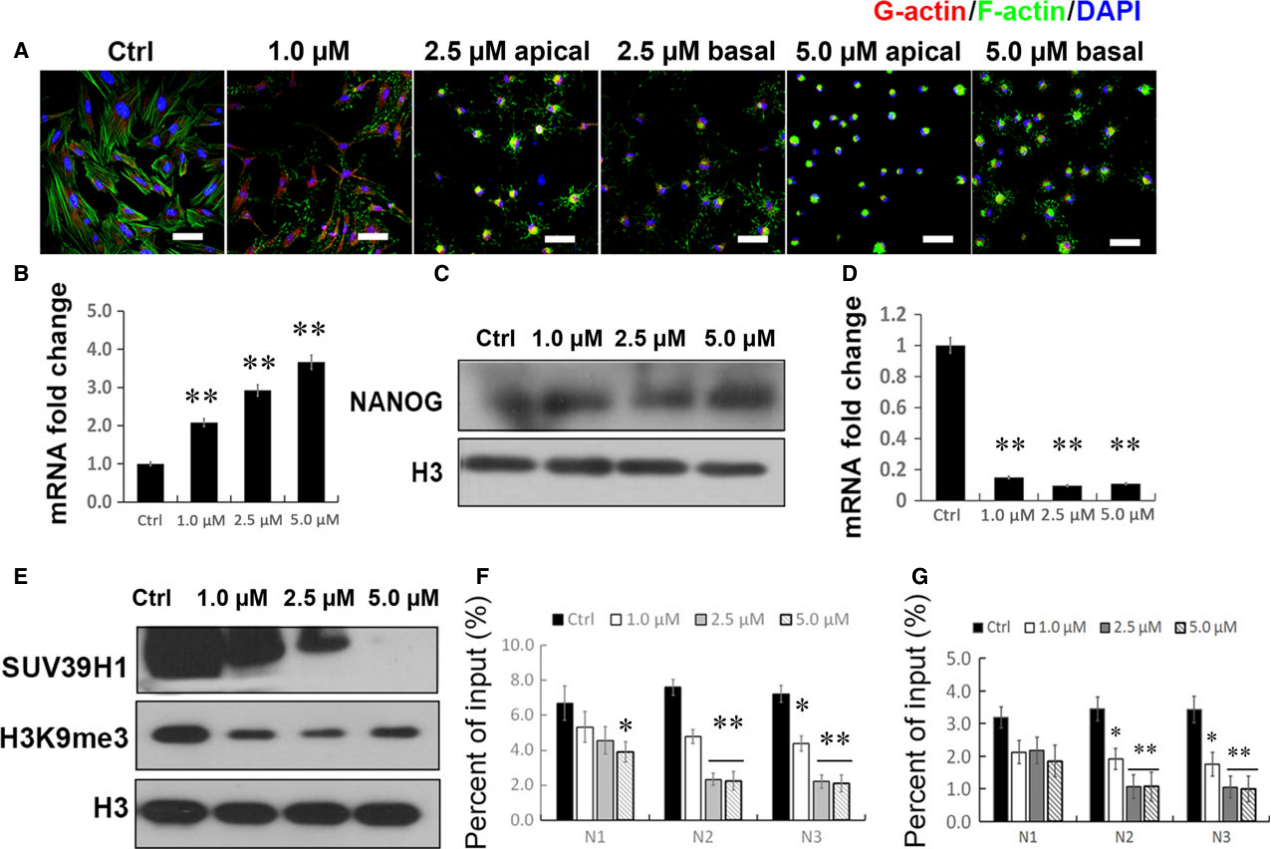
Released actin cytoskeleton promotes H3K9 demethylation and Nanog expression. Cultured MSCs at passage 6 in culture medium (DMEM+10% FBS) containing cytochalasin D at the indicated concentrations, and 0.1% DMSO as control for 60 hrs. (**A**) Fluorescence images of the F‐actin (green) and G‐actin (red) of MSCs. The cells get rounded with the addition of 2.5 and 5.0 μM cytochalasin D and presented different F‐actin pattern of apical and bottom layer (scale bar, 50 μm). Real‐time PCR was used to analyse the expression of Nanog (**B**) and Suv39h1 (**D**). Nuclear faction of MSCs was immunoblotted for Nanog (**C**), Suv39h1, H3K9me3 (**E**) and histone H3 (nuclear loading control). H3K9me3 (**F**) and Suv39h1 (**G**) ChIP‐qPCR assay was performed to evaluate the accumulation on Nanog. **P < 0.01 (*n* = 3), representative images of three independent experiments with similar results.

We examined if the tension of actin cytoskeleton was related to the up‐regulation of Nanog expression. Cytochalasin D, a commonly used cytoskeleton perturbing drug, was added into the MSCs culture. As a result, cytochalasin D induced a marked reduction in actin skeleton tension with rounded up cell morphology (Fig. 6A). Released actin cytoskeleton by cytochalasin D increased Nanog expression in a concentration‐dependent manner (Fig. 6B and C), together with Suv39h1 expression down‐regulation (Fig. 6D and E) and its abundance on Nanog promoter (Fig. 6G), which reduced the global level of H3K9me3 (Fig. 6E) and its accumulation on the promoter of Nanog (Fig. 6F). Moreover, considerable up‐regulation of pluripotent genes was observed in 3D MSCs (Fig. 5D, Fig. S2A), in line with previous studies 4, 10. However, only Nanog showed increase with cytochalasin D treatment in a concentration‐dependent manner (Fig. 6B, Fig. S2B).

## Discussion

### 3D culture down‐regulates integrin expression and releases actin cytoskeleton tension

Cells are intricately connected to the external environment through their cytoskeleton 29. Cell properties of shape, behaviour and even fate decisions can be influenced by several mechanic properties, including elasticity, geometry and adhesion, which ultimately impact cytoskeletal tension 36. Upon *in vitro* expansion, 2D MSCs become spread, flat and significantly increased in size 28, 37, 38. In line with these morphological changes, we found that the expression levels of integrin β1 in MSCs increased markedly upon culture expansion in monolayers 18. In this study, we further showed that MSCs in 2D culture exhibited branched and multiple‐directed F‐actin stress bundles at the cell edge as well as strengthened stress fibres transversing the cell body. The actin cytoskeleton was released in 3D MSCs, with very thin cortical actin filament outlining the cell. We supposed with the evidence above that 3D culture released the tension of cytoskeleton which was generated by stiff plastic substrate in conventional 2D culture, and thus induced morphological and mechanical changes in MSCs. Consequently, MSCs in 3D culture had more compact cytoplasm with approximately one‐fourth the volume of MSCs in 2D culture 4, 7, 16.

### Actin cytoskeleton force hampers MSCs stemness

Mesenchymal stem cells reside in a 3D niche composed of neighbouring cells and extracellular matrix in tissues. Apart from changes in substrate stiffness, 3D culture offers a condition which is more similar to that *in vivo*. A previous study showed that spheroid culture of MSCs increased their expression of E‐cadherin, and neutralization of the extracellular domain of E‐cadherin with blocking antibody inhibited spheroid formation of MSCs, suggesting that E‐cadherin plays a key role in spheroid formation of MSCs 20. Unlike the absence of E‐cadherin in MSCs in monolayer culture 10, N‐cadherin exists extensively in MSCs 39, 40. However, in monolayer culture, MSCs are usually cultured in low density to allow massive division, and the surface distribution of N‐cadherin, which mediated cell–cell contact, is mostly inhibited 41, 42. We found a marked increase of N‐cadherin expression in 3D MSCs, indicating massive cell–cell interaction establishment in 3D culture. The establishment of cadherin‐based tight junction facilitates enhanced signal exchanges between neighbour cells, which are indispensable in maintaining the pluripotency of stem cells 43, 44, 45.

Nanog is known for its critical role in the maintenance of pluripotency and fate determination of stem cells 46, 47. Nanog also sustains self‐renewal capability of MSCs and is down‐regulated in expression with culture expansion 48. Forced expression of Nanog reverses the effect of organismal ageing of MSCs 49. As we presented previously, the expression of Nanog increased under 3D culture 4, but the mechanisms are not clear. Actin cytoskeleton altered markedly in 3D culture. To examine the role of actin cytoskeleton stress tension in Nanog expression, we generated actin cytoskeleton relaxation in 2D‐cultured MSCs with cytochalasin D and found that the expression of Nanog increased, similar to that in 3D culture. Our data suggest that the aberrantly stretched and strengthened cytoskeleton in 2D culture interrupt Nanog expression, and the relaxation of stress fibre *via* 3D culture or pharmaceutical inhibition restores its expression.

### Actin cytoskeleton regulates the levels of Suv39h1 and H3K9me3

The expression of Nanog is associated with a loose chromatin configuration 50. H3K9 methylation enrichment in macro‐scale is the most pronounced chromatin change triggered by cultural environments 33, and its accumulation on the promoter region is the major barrier in the expression of core pluripotent genes in somatic cells 32, 34. Upon ESC (Embryonic stem cell) differentiation, the expression of Nanog decreased dramatically, accompanied with the accumulation of H3K9me3 on the region around the transcription starting site 51, 52. Suv39h1 is the most‐studied H3K9 tri‐methyltransferase 53, 54. Previous studies showed that knockdown of Suv39h1 rescued aberrant H3K9me3 accumulation 33, reversed cellular senescence 35 and increased iPS cell generation 32. Here, we showed that 3D culture of MSCs down‐regulated the expression of Suv39h1 significantly, accompanied with reduced occupancy of H3K9me3 in the promoter region of Nanog. Of note, a similar alteration was observed in the cells after cytochalasin D treatment, suggesting that the reduction of the H3K9me3 level is likely induced by the release of the actin cytoskeleton stress. In addition to the alteration in H3K9me3 level, we showed a marked difference in the nuclear distribution of H3K9m3, which was largely detected in the peripheral nucleus of 3D‐cultured MSCs, different from a central nucleus localization in 2D‐cultured MSCs. Our results suggest an important role of Suv39h1 and H3K9me3 in actin cytoskeleton‐mediated increase in Nanog expression in MSCs after 3D culture.

### Lasting nuclear F‐actin formation in 3D‐cultured MSCs

Previous studies suggest that nuclear assembly is crucially involved in gene transcription, but its role in MSCs has not been reported. The sponge‐like actin meshwork in Xenopus oocyte nuclei was the first reported F‐actin in the nucleus 55, 56. Filamentous actin formed when somatic nuclei were transferred into Xenopus oocytes, showing a role in the re‐activation of pluripotent genes in reprogramming 30. Using actin probes such as LifeAct, recent studies observed dynamic F‐actin formation in the nuclei of NIH 3T3 cells upon serum stimulation or during cell spreading 23, 31, which was essential in SRF‐mediated transcription.

In MSCs, the visualization of nuclear F‐actin had not been reported before. With LifeAct‐GFP‐NLS, we showed the existence of nuclear F‐actin in MSCs. In accordance with the previous observation in NIH 3T3 cells 31, cell spreading triggered dynamic formation of F‐actin in monolayer culture. Impressively, we found persistent existence of filamentous actin formation in the nucleus throughout the process of 3D culture, which looked like the actin meshwork in Xenopus oocyte nucleus. In monolayer‐cultured MSCs, the location and structure of the nucleus were restricted by actin cap, a bundle of apical stress fibres around nucleus 57, 58. Given the absence of tension in actin stress fibre in 3D MSCs, the persistent formation of nuclear F‐actin may provide a structural support to the rounded‐up nucleus in 3D MSCs. To the best of our knowledge, this is the first time to observe lasting nuclear F‐actin formation in somatic cells.

Compared with dynamic nuclear F‐actin formation triggered by serum stimulation or cell spreading, the nuclear actin meshwork in 3D MSCs was thicker. Given the essential role of nuclear actin meshwork in re‐activation of pluripotent genes 30, it is speculated that lasting nuclear actin formation in 3D MSCs may be involved in regulating chromatin organization 4, or in the re‐activation of pluripotent genes as reported in 3D MSCs 4, 10, 12.

Our study and others' indicate that 3D spheroid culture affect actin cytoskeleton arrangement. Several methods have been used to form cell aggregates such as hanging‐drop culture, suspension culture in low attachment plate, spinner flask and culture cells on special materials. It appears that MSCs undergo similar changes in actin cytoskeleton arrangement in aggregates generated by different methods 40, 59. F‐actin also showed a similar cortically lining arrangement in aggregates form by suspension culture 60 or microwells 61. In previous studies, cytoskeleton arrangement was mainly associated with MSCs differentiation switch, while here, we showed that the released actin tension induced Nanog expression, *via* the Suv39h1 mediated H3K9me3 on its promoter. Furthermore, we observed the F‐actin cytoskeleton both in cytoplasm and nuclear and found reduced and relaxed F‐actin in the cytoplasm while lasting formation of nuclear F‐actin in 3D culture MSCs. The connectivity between the nucleus and cytoplasmic actin may suggest an important mechanism in regulating cell activities, particularly for stem cells in tissues where they are in a 3D environment without over stretched actin cytoskeleton tension in cytoplasm.

## Conflict of interest

The authors declare that they have no competing interests.

## Supporting information


**Figure S1** β‐catenin in 2D and 3D MSCs.
**Figure S2** Pluripotent genes in 3D MSCs and cytochalasin D treatment.
