# Supplementary material for: 3D culture increases pluripotent gene expression in mesenchymal stem cells through relaxation of cytoskeleton tension
Source: J Cell Mol Med. 2017 Mar 9;21(6):1073–84. doi: 10.1111/jcmm.12946 (PMC5431137; doi:10.1111/jcmm.12946)
Supplement: Supplementary file 1 — Figure S1 β‐catenin in 2D and 3D MSCs. Figure S2 Pluripotent genes in 3D MSCs and cytochalasin D treatment. [file JCMM-21-1073-s001.docx]

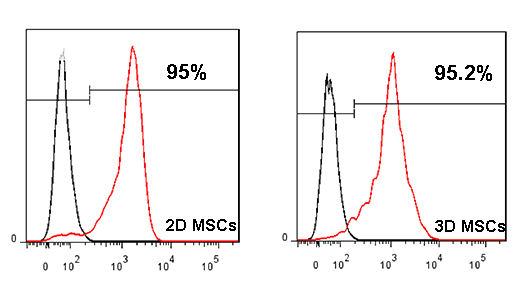


**Figure S1 β-catenin in 2D and 3D MSCs**

Expression of β-catenin in 2D and 3D MSCs quantified by flow cytometry.





**Figure S2 pluripotent genes in 3D MSCs and Cytochalasin D treatment.**

(A) Real-Time PCR analysis showed extensive up-regulation in pluripotent genes in 3D MSCs. (B) Real-Time PCR analysis of pluripotent genes in Cytochalasin D treatment with indicated concentration.**P<0.01(n=3)
