# Supplementary figures and images for: 3D culture increases pluripotent gene expression in mesenchymal stem cells through relaxation of cytoskeleton tension
Source: J Cell Mol Med. 2017 Mar 9;21(6):1073–84. doi: 10.1111/jcmm.12946 (PMC5431137; doi:10.1111/jcmm.12946)

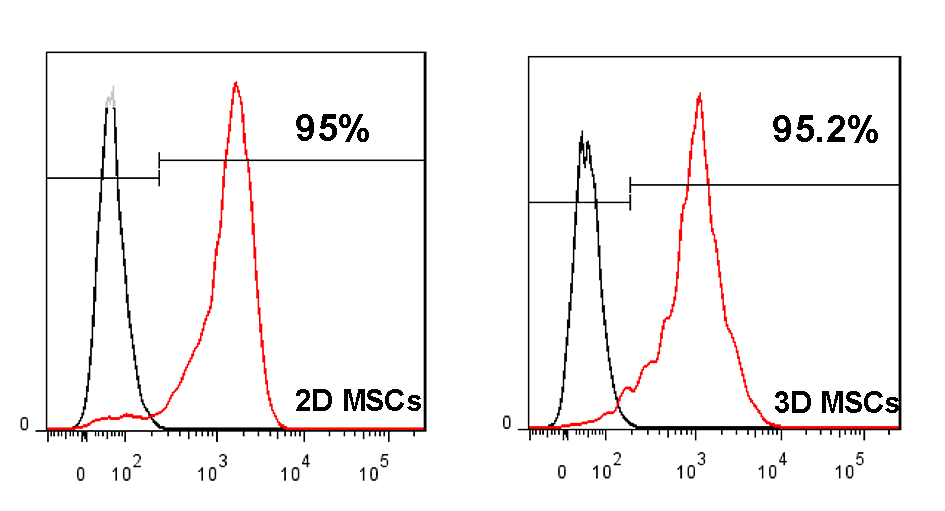

Supplement: Supplementary file 2 [file JCMM-21-1073-s002.tif]

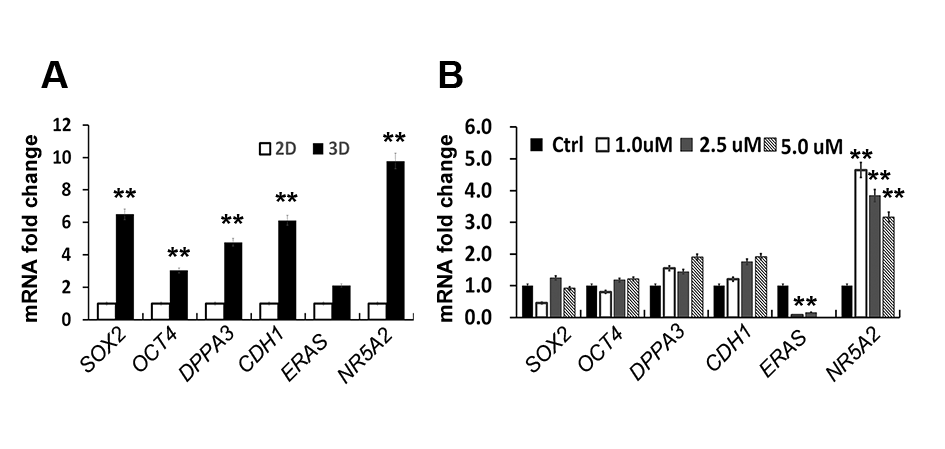

Supplement: Supplementary file 3 [file JCMM-21-1073-s003.tif]
